# Determinants of nurse satisfaction using insulin pen devices with safety needles: an exploratory factor analysis

**DOI:** 10.1186/s40842-015-0015-3

**Published:** 2015-11-09

**Authors:** Giovanni Veronesi, Carmine S. Poerio, Alessandra Braus, Maurizio Destro, Lavinia Gilberti, Giovanni Meroni, Estella M. Davis, Antonio C. Bossi

**Affiliations:** 1 Department of Clinical and Experimental Medicine, Research Centre in Epidemiology and Preventive Medicine, University of Insubria, Varese, Italy; 2 Metabolic Diseases and Diabetes Unit, A.O. Ospedale Treviglio-Caravaggio, P.le Ospedale, 1 – 24047 Treviglio, BG Italy; 3 Pharmacy Unit, A.O. Ospedale Treviglio-Caravaggio, Treviglio, BG Italy; 4 Medical Science Department, A.O. Ospedale Treviglio-Caravaggio, Treviglio, BG Italy; 5 Hospital Health Management Direction, A.O. Ospedale Treviglio-Caravaggio, Treviglio, BG Italy; 6 Creighton University School of Pharmacy and Health Professions, Omaha, NE USA

**Keywords:** Nurse satisfaction, Insulin therapy, Insulin pens, Safety needles, Inpatient care

## Abstract

**Background:**

A paucity of data exists to examine nurses’ satisfaction with the use of insulin pens with safety needles in hospitalized patients with diabetes. We investigated major determinants of nurses’ preference of the method of insulin administration in the context of a General Hospital in Northern Italy.

**Methods:**

Consecutive patients admitted to three hospital units of different care intensity requiring insulin received insulin therapy through either the vial/syringe method (October to December 2012) or pen/safety needles with dual-ended protection method (January to March 2013). Before the implementation of insulin pens, floor nurses received a specific training program for proper insulin pen injection technique including individual testing of the devices (pen/safety needles). At the end of the study, nurses completed the Nursing Satisfaction Survey Questionnaire. Cronbach’s alpha was used to determine the internal consistency and reliability of the questionnaire. Major determinants of satisfaction were investigated through an exploratory factor analysis. The association between each retained factor and time spent to teach patients how to self-inject insulin with pen devices was also investigated.

**Results:**

Fifty-three out of 60 nurses (mean age ± SD 36.2 ± 8.5 years, 85 % women, 57 % with 10+ years of working experience) returned the questionnaire. Internal consistency of the questionnaire was satisfactory (Cronbach’s alpha > 0.9). Three months after their introduction, about 92 % of nurses considered pen devices an “improvement” over the vial/syringe method. Two factors explained 85 % of nurses’ satisfaction, one related to convenience and ease of use, and the other to satisfaction/time spent for dose preparation and administration. The latter factor was inversely correlated with time spent on patients’ training tasks.

**Conclusions:**

Nurses’ satisfaction with pen devices was higher than previously reported, possibly reinforced by safety needles with dual-ended protection. Perceived workload was a major determinant of nurse satisfaction using pen devices with safety needles. To facilitate the introduction of insulin pens in the hospital setting, it should be specifically addressed during training programs in the switch-over period.

## Background

The prevalence of insulin pens worldwide has been recently estimated to be 60 % of insulin users; the figure being higher in Europe than in the US, and about 75 % in Italy [1]. Studies evaluating patient preference comparing self-administration of insulin using pen devices compared to traditional vial and syringe method found patients preferred insulin pens with respect to several items including ease of use, convenience, less injection pain, ease in handling, and ease of dosing [2]. Conversely, whether insulin pen devices should replace traditional vial and syringe in hospitalized patients is still a controversial subject [3]. Together with patients’ satisfaction [4, 5], economic evaluation [4–6] and safety issues related to the potential risk of biological contaminations for both nurses and patients [5–7], nurses’ satisfaction constitutes a key perspective for the management of hospitalized patients with diabetes requiring insulin injections. However, information on this topic is scarce, with the only data coming from the US, where 70 % of nurses considered insulin pens an “improvement” over conventional vial and syringe method 11 months after their introduction in two floors of one hospital [8]. The safety needles in this study differed from ours because they did not have a dual-ended protection. In addition, a limitation of this study was that nurses’ satisfaction was measured using a survey questionnaire developed by the authors that was not formally validated [8]. There are a variety of validated tools for patient’s satisfaction with self-administration of insulin [9–11], one of them specifically comparing pen devices with vial/syringe [11], but to the best of our knowledge, no validated questionnaire exists to assess nurses’ satisfaction with insulin administration method to hospitalized patients. The existence of a validated tool is important to be able to compare findings from different populations using a standardized measure, to understand the determinants of nurses’ satisfaction and utilize survey findings to adequately promote and enhance implementation of insulin pens in the hospital setting. In this paper, we report on nurse satisfaction, as assessed in Davis et al. [8], in the context of a pilot study aimed at implementing the use of insulin pens in hospitalized patients with diabetes at the Treviglio General Hospital in northern Italy. In addition, we performed an exploratory factor analysis, to investigate the latent structure behind nurses’ satisfaction.

## Methods

### Study setting

The SANITHY (SAfety Needles and Insulin pens at Treviglio Hospital – ItalY) study is a pilot study designed to implement the use of insulin pens in the hospital setting at the Treviglio General Hospital, northern Italy. From October to the end of December 2012, consecutive patients requiring multi-injection insulin therapy and admitted to three hospital units of different intensity of care (Cardiology and Coronary Care Unit; Neurology and Stroke Unit; Medicine and Urgency Unit) received the traditional vial and syringe method. Insulin pens and safety needles were adopted in the same hospital units the next successive three months from January to the end of March 2013 in consecutive patients requiring insulin therapy. The following insulin and prefilled insulin pens were utilized: Humalog^©^ and Humalog Kwikpen^©^ (Eli Lilly and Company, Indianapolis, USA) as rapid acting insulin; Lantus^©^ and Lantus SoloSTAR^©^ (Sanofi, Paris, France) as long-acting basal insulin. Together with pen devices, pen needles with a dual-ended protection safety system Autoshield Duo^©^ (Becton, Dickinson and Company, Franklin Lakes, NJ, USA) were utilized. The dual-ended protection covers both the portion of the needle in contact with the patient, and the back-end which penetrates into the rubber tip of the pen. Prior to the study, nurses received a specific training program on insulin pens consisting of small-group sessions and hands-on training, with individual testing to insure competence in using the insulin pen devices and safety needles properly. Thereafter, study nurses administered prescribed insulin therapy with pens and safety needles to inpatients, under an expert’s supervision, to demonstrate the acquired technical skill. Moreover, slides and a short explicative movie were available on our hospital Local Area Network portal (e-learning) and a 24 h, 7 day a week, toll-free phone number was active during the study period to interact with expert consultants. The pilot study was approved by the Independent Ethical Committee of the Treviglio Hospital.

### The nursing satisfaction survey questionnaire

The Nursing Satisfaction Survey Questionnaire (NSSQ) was proposed by Davis et al. to evaluate nurse satisfaction using pen devices as compared to vials/syringes in a sample of US nurses [8], in a study setting very similar to ours. The first section of the NSSQ collects information on the number of years practiced as a nurse, as well as on the previous experience with insulin administration and with study pen devices. Nurses’ satisfaction with insulin pen devices as compared to vial/syringes is then investigated through 8 items, each on a 5-point Likert scale ranging from “strongly disagree” to “strongly agree”, addressing different aspects such as insulin preparation and administration, convenience and ease of use, confidence and comfort in insulin administration, and time spent in dose preparation and administration. Items are reported in Table 2. Finally, the questionnaire attempts to quantify the time spent by the nurses to teach study patients how to self-inject insulin with each device, categorized as “<5 min”, “<15 min”, “<30 min”, “<60 min”, “60+ min”. One question is dedicated to naïve insulin patients, and another one to experienced insulin patients. The Italian version of the questionnaire is available upon request to the corresponding author.

### Study population and data collection

With the author’s permission, the NSSQ was translated to Italian and first administered to *n* = 44 nurses (questionnaire test sample) working in units not involved in the pilot study, but of the same Medical Sciences Department. The internal consistency was found to be satisfactory (Cronbach’s alpha = 0.91), thus, the questionnaire was administered to study nurses (*n* = 60, with characteristics comparable to the first group) at the end of the study period. The responses to the questionnaire were anonymous and completed independently.

### Statistical analysis

Study sample characteristics were summarized using standard statistics including mean, standard deviation and proportions. Responses to each of the 8 items assessing satisfaction with pen devices compared to vial/syringes were attributed a score ranging from 1 (“strongly disagree”) to 5 (“strongly agree”); the sum of the item responses could range between 8 and 40. We reported the mean score and standard deviation for each item, as well as the prevalence of a positive response defined as “agree” or “strongly agree”, as suggested by the authors [8]; and tested the null hypothesis of prevalence of positive answer equal to 50 % (i.e., no preference) using a two-sided exact binomial proportion test. To identify the latent structure of nurses’ satisfaction, we performed an exploratory factor analysis, given that the original NSSQ was not validated. The Kaiser-Meyer-Olkin measure of sampling adequacy value of 0.85 and the Bartlett test of sphericity (*p*-value: <.0001) supported the use of factor analysis. We fixed in 80 % the minimum proportion of cumulative variance to be explained by the factors as a general rule to decide the factors number; a scree plot was also used. Since the analysis made on the questionnaire test sample revealed a strong correlation between the 8 items, we considered an oblique factor rotation (promax), to allow for a non-zero correlation between the factors [12]. Internal validation for each retained factor was assessed through Cronbach’s alpha [13]. Finally, we assessed the relationship between each retained factor and time spent to teach patients how to self-inject insulin with pen devices. We reported the median (25°–75° percentile) of each factor score (as the sum of the responses to the items included in the factor) by categories of time, and formally tested the null hypothesis of no difference in factors score by time through a Kruskal-Wallis non parametric test [14]. All the statistical analyses were performed using the SAS software, version 9.3.2.

## Results

Out of the 60 study nurses, 53 returned the questionnaire, corresponding to a participation rate of 88 %. The study population of nurse respondents was on average 36 years old, 85 % women, 66 % had a nursing degree, and 57 % worked for 10 years or more as nurses (Table 1). Self-reported experience with insulin administration was extensive (50 patients or more) for 71 %, and 94 % had experience using insulin pens (mainly in outpatients settings) prior to the study. The BD AutoShield Duo^©^, new to the Italian market, had never been used by any of the nurses in the study. Most questionnaires were completed fully. Only 2 had questions that were left blank and were excluded from the analysis. Table 2 reports the mean score and the standard deviation, as well as the prevalence of response, to each question assessing nurses’ satisfaction with insulin pens over vial/syringes. On average, the total score for the sum of the 8 items was 34.6 ± 6.3, with a median of 38. Considering single items, the lower mean score was 4.0 for the item on dose accuracy (“Felt more confident I was giving the correct dose using pens”). This item was also the one with the lowest prevalence of positive answers (76.5 %, summing up 39.2 % of “agree” and 37.3 % of “strongly agree”). For the remaining items, the prevalence of positive answers was above 80 %, ranging from 84.3 % (item 7, time spent for insulin preparation and administration) to 92 % (items 3, convenience, 6, feeling comfortable, and 8, improvement). The *p*-values of the exact binomial test for positive answers different from 50 % were <0.0001 for all the items. The exploratory factor analysis suggested the existence of two factors to explain 85.3 % of total variance; the standardized regression coefficients identifying each factor are reported in Table 3. The first factor (items 6, 3, 8 and 4) was related to general aspects such as feeling comfortable with using pen devices, convenience, ease of use, and improvement over traditional method; this factor explained 77 % of variance. The second factor (items 5, 1, 2, 7) specifically focused on satisfaction in dose preparation and administration, including dose accuracy, and total time required for insulin injection; this factor explained 8.3 % of variance. The inter-factor correlation was positive and equal to 0.65; the Cronbach’s alpha assessing internal consistency for these two factors was above 0.90.

**Table 1:** Characteristics of the study population at the end of the study

N of responders	53
Mean age (SD)	36.2 (8.5)
Women (%)	85.4
Nursing degree (%)	65.9
Time practicing as a nurse (%)	
Less than 1 year	0.0
1 to 3 years	18.9
3 to 5 years	9.4
5 to 10 years	15.1
10+ years	56.6
Experience with insulin administration (%)	
None	0.0
Limited (up to 5 pts)	3.9
Average (up to 20 pts)	15.7
Substantial (up to 50 pts)	9.8
Extensive (50+ pts)	70.6
Experience using insulin pens (%)	94.3

*SD* standard deviation

**Table 2:** Mean score (standard deviation) and prevalence of response for each item assessing nurses’ satisfaction of insulin pens compared with traditional vial/syringe method. Responders with complete questionnaire (*n* = 51)

			% of nurses answering^b^				
#	Item description	Mean^a^ (SD)	Strongly disagree	Disagree	Unsure	Agree	Strongly agree
1	More satisfied with preparing insulin using pens	4.4 (1.1)	5.9	2.0	3.9	25.5	62.8
2	More satisfied with administering insulin using pens	4.5 (0.9)	2.0	3.9	3.9	27.5	62.8
3	Pens are more convenient	4.5 (0.8)	0.0	3.9	3.9	31.4	60.8
4	Pens are more simple & easy to use	4.3 (0.8)	0.0	3.9	7.8	41.2	47.1
5	Felt more confident I was giving the correct dose using pens	4.0 (1.1)	3.9	9.8	9.8	39.2	37.3
6	Felt more comfortable administering insulin to patients using pens	4.4 (0.8)	2.0	2.0	3.9	39.2	52.9
7	Took less time to prepare and give insulin using pens	4.1 (1.0)	2.0	9.8	3.9	43.1	41.2
8	Pens are an improvement over conventional	4.5 (0.7)	0.0	2.0	5.9	31.4	60.8
	Total score	34.6 (6.3)	-	-	-	-	-

^a^:Scoring: 1 = strongly disagree; 5 = strongly agree

^b^:response as in Davis et al. [8]

**Table 3:** Standardized Regression Coefficients, proportion of variance explained and internal consistency, for the first two factors explaining 80 % or more of variance of nurses’ satisfaction. Exploratory factor analysis with oblique rotation; responders with complete questionnaire (*n* = 51)

#	Item description	Factor 1	Factor 2
6	Felt more comfortable administering insulin to patients using pens	1.019	−0.123
3	Pens are more convenient	0.877	0.121
8	Pens are an improvement over conventional	0.803	0.185
4	Pens are more simple & easy to use	0.701	0.241
5	Felt more confident I was giving the correct dose using pens	−0.139	1.015
1	More satisfied with preparing insulin using pens	0.317	0.659
2	More satisfied with administering insulin using pens	0.415	0.597
7	Took less time to prepare and give insulin using pens	0.430	0.579
	Proportion of variance explained by each factor	77.0	8.3
	Factor’s internal consistency (Cronbach’s alpha)	0.94	0.92

During the study period, 38 and 47 nurses instructed patients how to use an insulin pen device to naïve or to experienced insulin users with diabetes, respectively. In Table 4 we report the median score (25°–75° percentile) for the two retained factors by time spent teaching patients, stratified by patient’s experience. When the number of patients was low (below 5), the original time categories were further collapsed to increase the size. In naïve insulin users, the factor score did not differ according to the different amount of time spent (Kruskal-Wallis p-values >0.05). Considering experienced insulin patients, the median scores for both factors decreased for increasing time spent. In particular, the median of the second factor decreased by 5.5 points from 19.5 to 14, as time spent ranged from below 5 min to above 16 min (Kruskal-Wallis *p*-value 0.04).

**Table 4:** Median score (25°–75° percentile) for the two factors retained from factor analysis according to different levels of time spent teaching a patient how to self-inject insulin with insulin pens, according to patient’s experience with insulin injection

	Naïve insulin user patients			Experienced insulin user patients		
	*n*	Factor 1^a^	Factor 2^b^	*n*	Factor 1^a^	Factor 2^b^
Time spent to teach how to use insulin pens						
<5 min	4	20 (16.5; 20)	18.5 (16; 20)	20	20 (17; 20)	19.5 (17; 20)
<15 min	24			20	19 (16; 20)	18 (16; 19)
<30 min	9	19 (16; 20)	19 (16; 19)	2	17 (14; 19)	14 (8; 19)
31+ min	1			5		
Kruskal-Wallis test *p*-value	-	0.6	0.3	-	0.09	0.04

When the number of patients was low (below 5), the original time categories were further collapsed to increase the size

^a^:sum of responses to the following items: feeling comfortable, convenience, improvement, ease of use (item 6, 3, 8, 4). 1 = strongly disagree; 5 = strongly agree

^b^:sum of responses to the following items: satisfaction in dose preparation and administration (item 5, 1, 2, 7). 1 = strongly disagree; 5 = strongly agree

## Discussion

In this study population of 53 hospital nurses completing a pilot study on insulin administration using prefilled pens and BD Autoshield Duo^©^, about 92 % of nurses considered these devices an improvement over traditional vial/syringe method. Two factors explain 85 % of nurses’ satisfaction, one related to feeling comfortable, convenience, ease of use and improvement; while the other focused on satisfaction in dose preparation and administration, including time spent, and confidence in dose accuracy. The latter factor only was inversely associated with time spent teaching patients how to self-inject insulin using a pen device. A growing body of evidence shows several benefits of insulin pens for inpatient care, both from the hospital’s and the patient’s perspectives. Insulin pens have being suggested to be cost-effective and to improve patients’ quality of life during the hospital stay as well [4, 5, 15]. However, information on nurses’ satisfaction using insulin pens in hospitalized patients is sparse, with the only data coming from the US [8]. The nurse level of satisfaction with insulin pens, measured with the same questionnaire, from our study was generally higher than in Davis and colleagues [8], who reported a positive answer for the item “improvement” in 70 % of nurses (92 % in our population). Two reasons may explain these differences. First, we assessed satisfaction at 3 months while Davis et al. after 11 months of insulin pen use, suggesting that satisfaction may wane over time if not adequately supported and promoted. Second, although both studies utilized insulin pen safety needles to reduce the risk of needlestick injuries [16], the BD Autoshield Duo^©^, used in our study only, had a dual ended protection. Thus, it is possible that the consciousness of a complete protection from needle-stick injuries played a major role in reinforcing nurses’ satisfaction. Training alone, in fact, can reduce, but not eliminate, the risk of injuries related to pen use [6]. The population of nurses caring for patients with diabetes is subject to a relatively high risk of needle-stick injuries, with a significant amount of post-injury emotional distress [17]. Study nurses experienced 2 needle-stick injuries during the 3 month-period with standard syringe/vial for insulin administration, and no injury in the experimental period with pen devices and double-safety needles (data not shown). Strengths of this study include that study participants represented nurses from three hospital units with different care intensities in a general hospital (Cardiology and Coronary Care Unit; Neurology and Stroke Unit; Medicine and Urgency Unit), a high survey response rate (88 %), and the very satisfactory internal consistency in the questionnaire’s compilation (Cronbach’s alpha >0.90). A study limitation was the short time period of insulin pen and safety needle use (3 months) from which to evaluate nurses’ satisfaction with the new insulin administration method. Other factors may contribute to lower nurse satisfaction with insulin pen devices over a longer time period. For instance, to minimize the risk of contamination related to sharing the pen device among multiple users [7], study nurses performed a “sure self-identification” by asking name, surname, and date of birth to every patient. A more sophisticated electronic “code-number” system of identification could be more suitable over the long-term period [18]. We also recommend caution when generalizing our findings to nurses working in non-medical hospital units such as surgery or emergency departments. Finally, although our sample size meets some minimum requisite for exploratory factor analysis (cases/item ratio > 5), it is desirable that our findings should be replicated by larger confirmatory studies. To the best of our knowledge, our exploratory factor analysis is the first attempt to identify major determinants of nurses’ satisfaction using insulin pen devices with safety needles. The first factor to a great extent matches patients’ preference for pen devices over the traditional vial/syringes in terms of ease and convenience of use [2]. The second factor instead could be interpreted as nurses’ perception of work load, as it referred to several aspects of dose preparation and administration, including perceived time spent, and confidence in dose accuracy. This factor was also inversely related to time spent teaching patients how to self-inject insulin using pen devices. Nurses’ training programs for the implementation of pen devices in the hospital setting are mainly focused on reducing risk to the patient and personnel [6, 18, 19]. Our findings imply that nurses’ satisfaction can be strengthened during the training program by targeting all aspects related to nurses’ perceived workload. The proposed educational program for instance included several supervised interactive sessions, with individual competency testing of insulin pens with dual-ended safety needles with additional instructional material available on-line (e-learning). Moreover, familiarization with insulin pens in the hospital could reduce future costs in the outpatient setting [3]. Appropriate discharge education should be provided to patients who transition to insulin pen devices from the vial/syringe method.

## Conclusions

In conclusion, this study confirmed nurses’ preference for pen/safety needles over the traditional vial/syringes method for insulin administration in the setting of a General Hospital in Northern Italy. The recent introduction of a safety needle with a dual-ended protection may have strengthened satisfaction compared to previously published data. Nurses’ workload perception was a determinant of satisfaction, and should be targeted during an interchange training program for successful implementation of insulin pens in the hospital setting.
